# Unveiling the underlying structure of awe in virtual reality and in autobiographical recall: an exploratory study

**DOI:** 10.1038/s41598-024-62654-3

**Published:** 2024-05-30

**Authors:** Alice Chirico, Francesca Borghesi, David B. Yaden, Marta Pizzolante, Eleonora Diletta Sarcinella, Pietro Cipresso, Andrea Gaggioli

**Affiliations:** 1 Department of Psychology, Research Center in Communication Psychology, Università Cattolica del Sacro Cuore, Milan, Italy; 2 Department of Psychology, University of Turin, Via Verdi 10, 10124 Turin, Italy; 3 Department of Psychiatry and Behavioral Sciences, Johns Hopkins University School of Medicine, Baltimore, MD USA; 4 IRCCS Istituto Auxologico Italiano, Milan, Italy

**Keywords:** Virtual reality, Awe, Autobiographical recall, Emotion, AWE-S, Exploratory factor analysis, Confirmatory factor analysis, Psychology, Human behaviour, Statistics

## Abstract

Over the last two decades, awe has attracted the attention of an increasing number of researchers. The use of virtual reality has been identified as one of the most effective techniques for eliciting awe, in addition to more personalized methods for inducing emotion, such as autobiographical recall. However, previous measures of awe were unable to uncover the hidden structure of this experience. Awe experience scale (AWE-S) has been validated as a comprehensive measure of contingent awe in English, providing new opportunities for analysis. In this two-phases study, we investigated whether the latent structure of the experience of awe evoked by the autobiographical recall technique (Study 1) overlapped with that induced by exposing participants to a validated virtual reality awe-eliciting training (Study 2). The original English AWE-S structure held both in autobiographical recall induction and virtual reality-based elicitation. Despite evidence of overlap between English and Italian structures, low correlations were found between Italian trait measures used to test the concurrent validity of the AWE-S in the Italian sample and AWE-S state dimensions. This study highlights cultural differences in awe experience, trait, and state variations, and provides new insights into the standardized induction of this emotion through simulated environments.

## Introduction

*The fairest thing we can experience is the mysterious. It is the fundamental emotion which stands at the cradle of true art and true science. He who knows it not and can no longer wonder, lo longer feel amazement, is as good as dead, who can no longer pause to wonder and stand rapt in awe, is as good as dead: his eyes are closed.*

Albert Einstein in "*The World as I See It*”.

It may be not surprising that, after centuries of silence, there is now a great deal of scientific debate in the field of psychology concerning of the multifaceted and mysterious emotion of awe^1–7^, which dwells on the “*upper reaches of pleasure and on the boundary of fear*”^1^ (p. 297). The reasons for this renewed interest and controversy are various. First, there is a tendency to exaggerate the beneficial effects of this emotion (https://www.newscientist.com/article/mg23531360-400-awesome-awe-the-emotion-that-gives-us-superpowers/, accessed on 1 July 2023.) theoretically, and empirically. While awe’s neuropsychophysiological profile is still under study^8,9^ (e.g., as a result of both positive and negative experiences of awe, the left middle temporal gyrus was deactivated, while only positive awe resulted in increased functional connectivity in these areas and in the anterior/posterior cingulate cortex^10^), a growing number of studies has demonstrated a link between this emotion and overall mental, physical health^11^ as well as wellbeing^12^. Awe has a number of interesting subjective qualities, as it has been shown to expand time perception^13^, diminishes the sense of self ^14^, induces uncertainty^15^, and fosters prosocial intentions, attitudes, and behaviors in adults^14,16,17^ and in children^18^. Awe has been shown to promote a sense of connectedness with nature^19,20^ and with all humans^21^, along with intention to safeguard the natural environment^22^. Crucially, these awe-related patterns emerged in a similar way in eastern as well as in western cultures^21^.

However, the exact definition of awe is still a matter of contention. Among other valuable definitions of awe (e.g., ^23–28^), Keltner and Haidt’ seminal and multidisciplinary account of awe^1^, which has often been cited as the basis for the experimental induction of this emotion^29^, distinguished two main dimensions of this emotion. Awe arises from stimuli so vast (i.e., *vastness*) to require an accommodation of individual’s mental frames^1^ (i.e., *need for accommodation*) (or their expansion^14^ due to exceeded expectancies^22^). Keltner and Haidt suggested that awe has various ‘flavors’^1^ (e.g., admiration, elevation, the sublime).

Awe has typically been measured using single items (e.g., “I felt awe”), which motivated the development of a multi-dimensional state measure of awe^23^. The validation studies of the first standardized questionnaire for measuring awe, the Awe Experience Scale^23^, revealed the multi-dimensional latent structure (p. 1) of this experience^23^. Some typical features of emotions emerged, such as the presence of a distinct expressive pattern (i.e., *Physical sensations*) as manifested in goosebumps, as well as facial expressions (eyes widening, mouth opening). This set of studies also outlined three other “facets” of this emotion, typical of a self-transcendent experience^24^, such as an alteration of time (i.e., *Time* dimension), a sense of self-diminishment (i.e., *Small self*), and of connectedness (i.e., *Connectedness*). Often, awe has been labelled as a self-transcendent emotional experience^24–28^. Crucially, self-transcendent properties of awe have already been shown to be connected to positive outcomes such as to inspiration^30^, authenticity^31^, and prosocial behaviors^32^.

The AWE-S scale has been validated in English and with mostly US participants^33^. However, it has not been tested psychometrically in Italian and rarely in other non-US populations^34^. Evidence suggested that there can be cultural differences in appraisals leading to emotions and in emotion regulation strategies and emotional expressions between US and Italian participants (e.g.,^35–37^) . Moreover, Italy differs from US at several levels. Despite they can be both considered as individualistic cultures^38^ Italy belongs to Latin Europe, and it has peculiar linguistic backgrounds, which can be traced back to the Latin and the Romance language. In line with this, some key differences have been already found regarding basic emotions words equivalence in English-speaking countries vs. Italy^39^. For instance, studies conducted on Italian language, have showed that intensity of a given emotional experience predicted the degree of perceived prototypicality of an emotional lexical term^40^. Italy has a rich history concerning the sublime—as a proximal experience to awe^41^,especially in the philosophical discourse, and disentangling the prototypical nature of these two phenomena is an urgent and still open issue^41,42^. This is far more crucial if considering the negative nuances of awe experience (i.e., the sublime emphasizes a fear component) and that recent evidence suggested a mixed emotional nature of threat-based version of awe, quite different from classical induction of positive awe experiences^43^. Specifically, threatening awe has been shown to produce adaptive outcomes for the individual. This is evidenced in threatening awe’s ability to promote prosocial tendencies^13^, and the involvement of brain areas associated with empathy and self-reflection as presented in^15^. Therefore, more studies on the psychometric properties of existing language-based self-report US measures of affects and emotions are needed.

Complicating matters further, empirical, and theoretical evidence have progressively showed that there may be also different “versions” of awe^44^. For instance, there is, at least, an every-day awe, which is generally conceived as a positive emotion^45^ similar to wonder^46^. In this regard, usually, awe is framed and studied as a distinct positive emotion characterized by specific behavioral, physiological and cognitive patterns^11,47,48^. A more intense kind of awe experience may also exist^49^, which is closer to a transformative phenomenon imbued with a blend of positive and negative emotional components and philosophically rooted into the notion of the sublime^41^. According to a recent quantitative correlational study based on US-standardized awe measures and analyzing the relationship between the sublime and awe, showed several positive correlations between these two phenomena, especially concerning the self-transcendence domain^5^, thus, suggesting a certain degree of overlap between the two concepts. Increasingly, it has been proposed that awe should be conceived more as an experience rather than just as a typical emotion^50–52^. Qualitative accounts of this emotion^25,53^ reinforced this assumption by revealing other facets of awe, such as ineffability, the numinous, or heightened perception.

Methodological advancements have been proposed to move towards a more ecologically valid analysis of this emotional experience. Accordingly, recent studies have begun to combine phenomenological approaches, neuropsychophysiological measures, and Virtual Reality (VR)^6,54^. Several recent studies have used Virtual Reality (VR) as a novel paradigm for studying even complex or paradoxical experiences of awe in the lab^12,55,56^. These methods allow researchers to move from a low intensity form of awe to a more profound version of it, which enables more self-transcendent kinds of experiences^57–59^.

Reproducing intense instances of awe in the lab depends on the combination of a specific medium with relevant content^60^. Specifically, it was the combination of VR with awe-inspiring content that effectively triggered profound experiences of this emotion. That is, content design matters, even if not alone. Such methods allow researchers to progress beyond merely showing videos on a computer screen, which previous awe research relied on in many studies.

Another open question about awe concerns how a range of situations and stimuli evoke awe. When participants were asked to recall personal experiences of awe, nature emerged as one of the most frequent elicitors^23^, followed by great skills, an encounter with God, great virtue, monument, powerful leader, grand theory, music, art, and epiphany. However, curiously, there were also several other frequent “non classified” elicitors, which emerged from the English validation of the Awe Experience Scale; among them, authors mentioned childbirth^23^. Recently, Keltner and colleagues proposed to group domains able to evoke awe in to the following categories: spiritual engagement, music, dance, and psychedelics^11,46^. Recently, also collective inductors of awe have been studied and proved effective in eliciting fairly intense moments of awe^3^. Specifically, at the experimental level, awe has been reliably elicited across different methodologies, also showing unique effects on study outcomes relative to other positive emotion states^28^.

Research on awe could be divided into studies focused on low intensity “everyday” awe, at the dispositional^25,61,62^ and at the state level^61^ and, on the other hand, those pursuing the goal of capturing high intensity moments of awe^63–65^ such as (but not necessarily limited to) VR^6,66^. Across all of these studies, the main dimensions of awe (*vastness* and *need for accommodation*) are generally generated even across quite different emotion-induction techniques^29^, but a core issue remains: does the multidimensional latent structure of awe change across induction techniques and levels of intensity?

In the present studies, the primary interest was first to create an Italian version of the AWE-S and then to examine the factor structure of the AWE-S after two different methods of awe induction. On the one hand, we relied on the classical method of autobiographical recall to elicit awe, by asking participants to recall a personal experience of awe. On the other hand, another group of participants was exposed to awe-inspiring virtual reality scenarios, as a standardized methodology of emotion induction. The aim was to investigate to what extent the latent structure of the experience of awe elicited by the autobiographical recall overlapped with awe induced by the standardized exposure to awe inspiring VR, in two different studies. In Chirico^67^, it has been suggested that different awe-inducing techniques may result in different levels of intensity of awe, since the quality of each emotional experience can be affected by the different emotional induction techniques used^68^.

In this set of two studies, we analyzed the latent structure of the global experience of awe by administering the Awe- Experience Scale (AWE-S) to a sample of Italian individuals, asking them to recall an awe-inspiring moment of their life (i.e., autobiographical recall) and administering the AWE-S (study 1). Then, we tested if the resulting model was held with the experience of awe induced in VR (study 2). We then inspected the factor structure of an Italian version of the AWE-S.

## Study 1

### Materials and method

#### Participants

The study included 350 participants who all voluntarily took part in the study (255 females—mean age = 31.8; S.D. = 14.7; 95 males − mean age = 29.9; S.D. = 15,4). They were adults over 18, mostly educated, with an average of 14 years of schooling (18.9% n = 61 of them had a middle school diploma, 32.9% n = 115 presented a high school diploma and 22.9% n = 80 had a master’s degree. They lived mostly with their family (74.4%, n = 257), and half of them were Christian Catholic (54%, n = 189): the other half did not express their religious orientation. Their families were mostly Christian Catholic (83.7%, n = 293).

Participants were recruited through announcements on principal social network platforms, such as email, Facebook, and Instagram. People who showed interest in the study received a link provided with a description of the main objective of the research, containing all the questionnaires that they were required to fill in to participate in the research. The survey was conducted entirely online using Qualtrics, a secure online survey distribution and data collection program.

The experimental protocol was approved by the Ethical Committee of the Università Cattolica del Sacro Cuore prior to data collection. Each participant provided an electronic informed consent for study participation. Participants’ consent and all methods were carried out in accordance with the Helsinki Declaration.

#### Measures

##### Italian version of the awe experience scale (AWE-S)

The Awe Experience Scale^23^ is a self-report questionnaire assessing the intensity of state awe lived by participants during a peculiar event. Participants were asked to recall and briefly describe an event from the past in which they experienced awe. The AWE-S questionnaire contains 30 items on a seven-point Likert scale (1 = Strongly Disagree, 2 = Moderately Disagree, 3 = Somewhat Disagree, 4 = Neutral, 5 = Somewhat Agree, 6 = Moderately Agree, 7 = Strongly Agree). It consists of six main dimensions (Factors): *Vastness*; *Need for Accommodation*; *Connectedness*; *Self-diminishment*; *Physical Sensations*; *Time*. Two bilingual translators translated the original English items into Italian and then back translated them into English in order to ensure the most accurate translation possible and avoid conceptual or linguistic errors. Their translations were checked at the semantic, lexical, and syntactical level following guidelines provided by Borsa et al^69^.

Other self-report instruments for the assessment of contingent experiences of awe have also been developed^59–61^. However, in this study, we chose to administer the AWE-S as a widely used scale for measuring awe and in a more robust and granular way. The Italian translation of the specific term *awe* by consultation among the two bilingual experts and authors gave rise to a periphrasis in which a noun and an adjective were combined (it. *profonda meraviglia*)^70^, that is, “profound” and “wonder”, hinting at something more intense and complex than wonder itself.

##### Italian validated version of the positive and negative affect scale (PANAS)

The PANAS represents positive and negative affective experiences^63^. In this study, we adopted the Italian validation^64^ of this questionnaire consisting of 20 adjectives measuring two dimensions of affective experience: positive (10 adjectives) [Positive Affect scale (PA)] and negative (10 adjectives) [Negative Affect scale (NA)]. This study adopted the state version by instructing participants to indicate to what extent each of the 20 adjectives describes the way they felt while recalling the awe experience on a 5-point Likert scale (1 = strongly disagree to 5 = strongly agree). Awe has been generally considered as a positive emotion^71–73^, thus, measures tapping into negative affect dimension should act as divergent ones.

##### Italian validated version of the dispositional positive emotions scale (DPES)

In this study, the Italian validation^74^ of the Dispositional Positive Emotions Scale^71^ was used. It is a self-report measure assessing the disposition to live the following positive emotions: Happiness; Compassion; Amusement; Love; Pride; Awe. The scale requires participants to answer 37 items rated on a 7-point Likert scale, anchored at 1 = Strongly disagree and 7 = Strongly agree.

The Italian Dispositional Awe scale showed moderate positive correlations with the Positive Affect Factor^65^, and negatively with the Negative Affect factor. By relying on this evidence, we considered the PA Scale as a convergent validity measure and the NA as a divergent one.

##### Italian version of emotion regulation questionnaire (ERQ)

The Emotion Regulation Questionnaire^75^ is a validated 10-item questionnaire on a 7-point Likert scale (1 = strongly disagree; 7 = strongly agree) aiming to measure two strategies of emotion regulation: Cognitive Reappraisal (Factor 1: 6 items) and Expressive Suppression (factor 2: 4 items). In the current study we used the Italian version of the ERQ^76^. Participants were required to think about how they usually regulate their own emotions. Based on the results of a study focusing on the Italian validation of the DPES, this was used as a measure of convergence and divergence validity. This study found that dispositional awe was negatively correlated with Suppression (but not significantly) and significantly positively correlated with Reappraisal.

#### Procedure

The survey was conducted using Qualtrics survey software and an online informed consent form was provided to participants. There were no minors included in the study. Participants were recruited through flyers, social networks, and word-of-mouth. Participants were told that they would be asked to complete a questionnaire regarding awe. Then, they filled the first part of the questionnaire to gather socio-demographic data and completed the Italian translation of the AWE-S. Participants were invited to recall a personal experience of awe using the same instructions from Yaden et al.^23^ Then, participants answered the 30 items of the Italian AWE-S. Finally, participants completed the Italian version of the DPES, PANAS, ERQ.

## Results

### Data analyses

We carried out an Exploratory Factor Analysis (EFA) on 30 items of the Italian version of the AWE-S, in order to investigate the latent factorial structure of the Italian version vs. the original English one. EFA was performed using Jamovi statistic software version 2.3.21. Then, several Pearson’s correlations were carried out to test convergent and divergent validity of the Italian version of the AWE-S.

### Preliminary data analysis

No missing cases were found for AWE-S and for the other scales. Thus, a final sample of 350 participants was used. To check the normality of the scale, we examined skewness and kurtosis values of each item. As in the validation of the original scale, it was expected some values would not follow a normal distribution, because the prompt asked for a particularly intense awe-inspiring experience. In line with this, item 16, 17, 18, 20 featured high levels of kurtosis. In the original English version of the scale these items converged in the factor of *Vastness*, so it is plausible that these values depend on the intense extraordinary nature of this recalled experience.

**Figure 1:**
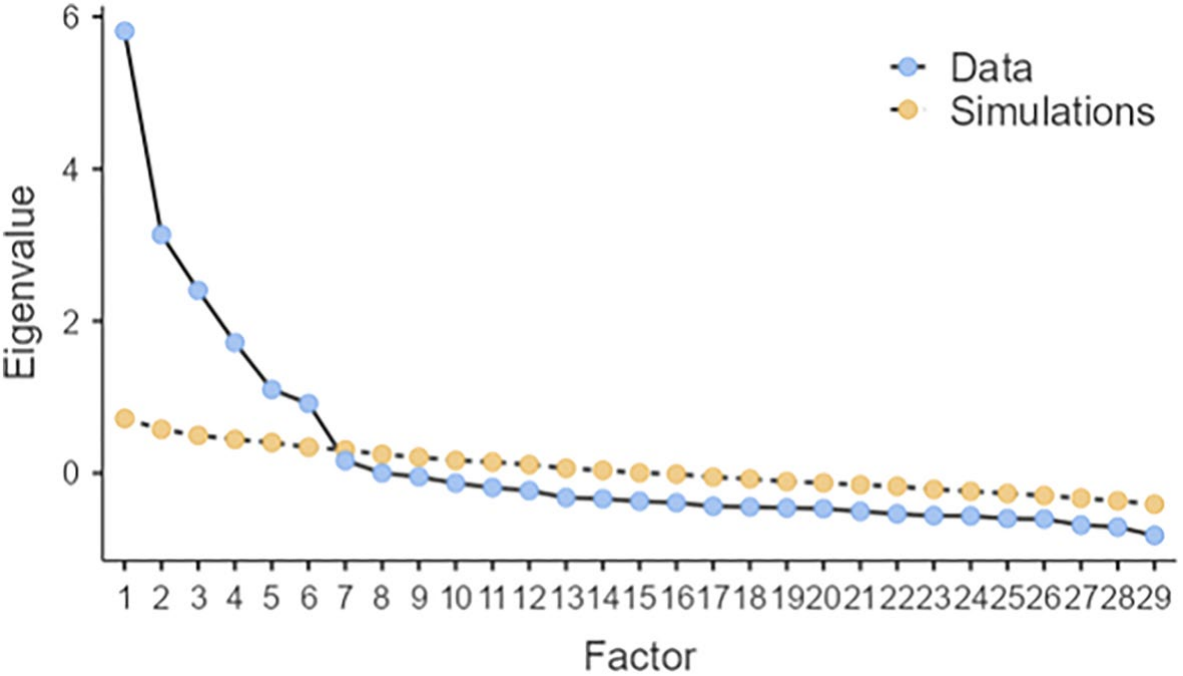
Parallel Analysis: Scree Plot. 6 factor solution.

To examine the structure of the Italian AWE-S, an EFA analysis was conducted out on the original set of items. Prior to performing the EFA, a parallel Monte Carlo simulation analysis^70^ was run on the 30 items to determine the optimal number of factors to retain (Fig. 1). This analysis indicated a maximum of a six-factor structure. The six-factor solution was further validated by the eigenvalues, indicating that 6 factors had eigenvalues greater than 1.0, collectively accounting for 61% of the total variance. Additionally, an inspection of the correlation matrix revealed that all coefficients were 0.50 and higher.

### Exploratory factor analysis

Therefore, we chose to test the 6-factor solution in line with the original structure of the scale, as suggested by Parallel Analysis. Moreover, given the nature of the scale, which assesses dimensions of the same experience, we expected that our factors would be correlated. Therefore, we carried out a Principal Axis Factoring (PAF) analysis with promax rotation. The 6-factor solution explained 61% variance.Keiser Meyer Olkin (KMO = 0.834) and Bartlett’s test of Sphericity [χ2 (425) = 6625; *p* < .01] on the rotated data matrix supported this solution. In this factorial solution, item 29 *(Italian translation: Ho fatto fatica a capire fino in fondo tutto ciò che stavo sperimentando in una volta sola)* did not load on any factor and showed low communality. Therefore, item 29 was dropped, and the 6-factor solution provided the maximum number of stable and reliable factors. Factor order reflects the original English version. Therefore, even though “Self-loss” was extracted as the first factor since it explained the highest portion of variance, we chose to label it as “Factor 2” for clarity, to adhere to the original order of items in the English version (Tab.1).

**Table 1:** Final factor solution: Pattern matrix showing factor loadings from exploratory factor analysis PAF with promax rotation in the final version of the scale (29 items, n = 350).

	Time	Self loss	Connection	Vastness	Physical sensations	Need for accommodation
	1	2	3	4	5	6
1. Ho percepito le cose rallentare momentaneamente (I sensed things momentarily slow down)	0.738					
2. Ho notato che il tempo stava rallentando (I noticed time slowing)	0.808					
3. Ho sentito cambiare la mia percezione del tempo (I felt my sense of time change)	0.796					
4. Ho sperimentato il trascorrere del tempo in modo diverso (I experienced the passage of time differently)	0.681					
5. Ho avuto la sensazione che un momento durasse più a lungo del solito (I had the sense that a moment lasted longer than usual)	0.816					
6. Ho sentito che il mio senso del sé era diminuito (I felt that my sense of self was diminished)		0.962				
7. Ho sentito il mio senso del sé rimpicciolire (I felt my sense of self shrink)		0.831				
8. Ho sperimentato un senso del sé ridotto (I experienced a reduced sense of self)		0.952				
9. Ho sentito il mio senso del sé diventare in qualche modo più piccolo (I felt my sense of self become somehow smaller)		0.977				
10. Mi sono sentito piccolo rispetto a tutto il resto (I felt small compared to everything else)		0.649				
11. Ho avuto la sensazione di essere connesso a ogni cosa (I had the sense of being connected to everything)			0.761			
12. Ho sentito un senso di comunione con tutti gli esseri viventi (I felt a sense of communion with all living things)			0.919			
13. Ho sperimentato un senso di unità con tutte le cose (I experienced a sense of oneness with all things)			0.595			
14. Mi sono sentito strettamente connesso con il genere umano (I felt closely connected to humanity)			0.787			
15. Ho avvertito un senso di completa connessione (I had a sense of complete connectedness)			0.489			
16. Ho sentito di essere in presenza di qualcosa di grandioso (I felt that I was in the presence of something grand)				0.596		
17. Ho sperimentato qualcosa di più grande di me (I experienced something greater than myself)				0.723		
18. Mi sono sentito in presenza della grandiosità (I felt in the presence of greatness)				0.695		
19. Ho percepito qualcosa che era molto più ampio rispetto a me (I perceived something that was much larger than me)				0.671		
20. Ho percepito la vastità (I perceived vastness)				0.653		
21. Ho sentito la bocca spalancarsi (I felt my jaw drop)					0.636	
22. Mi è venuta la pelle d’oca (I had goosebumps)					0.826	
23. Sono rimasto senza fiato (I gasped)					0.717	
24. Mi sono venuti i brividi (I had chills)					0.757	
25. Ho sentito gli occhi spalancarsi (I felt my eyes widen)					0.63	
26. Mi sono sentito in difficoltà nell'elaborare mentalmente ciò che stavo sperimentando (I felt challenged to mentally process what I was experiencing)						0.713
27. Ho trovato difficile comprendere l’esperienza pienamente (I found it hard to comprehend the experience in full)						0.949
28. Mi sono sentito in difficoltà nel comprendere l’esperienza (I felt challenged to understand the experience)						0.945
29. Ho cercato di comprendere la grandezza di ciò che stavo sperimentando (I tried to understand the magnitude of what I was experiencing)						0.652

Items corresponding to each factor were summed. The final Italian AWE-S, therefore, included 29 items total, with 5 items per factor (despite for Factor 6, with 4 items). Each factor was tested with reliability Cronbach’s alpha (α), showing high statistic reliability. Standardized alphas were as follows: (F1) Time α = 0.87 (F2) Self-loss α = 0.94; (F3) Connection α = 0.85; (F4) Vastness α = 0.80; (F5) Physical Sensations α = 0.84; (F6) Need for Accommodation α = 0.88.

### Concurrent validity

At this stage, the correlation between factors resulting from EFA and other constructs found in validated scales was analyzed. We conducted initial validation of the AWE-S by with respect to the trait scale of the Dispositional Positive Emotion Scale (DPES), the Italian version of the PANAS, and the ERQ (Table 2).

**Table 2:** Pearson’s correlations among DPES, PANAS, ERQ and AWE-S Factors: Significant correlations are in [bold].

	Time	Self loss	Connection	Vastness	Physical Sensations	Need for accommodation	Negative affect	Positive affect	Happiness	Compassion	Amusement	Love	Pride	AWE	Reappraisal
Time	–														
Self loss	0.23***	–													
Connection	0.281***	0.08	–												
Vastness	0.248***	0.323***	0.375***	–											
Physical sensations	0.269***	0.096	0.139**	0.201***	–										
Need for accommodation	0.245***	**0**.234***	− 0.061	− 0.012	0.09	–									
Negative affect	0.002	**0**.132*	− 0.150**	− 0.129*	− 0.04	0.293***	–								
Positive affect	0.011	0.039	− 0.028	− 0.031	− 0.025	0.154**	0.735***	–							
Happiness	0.115	− 0.184*	0.178*	0.03	0.096	− 0.135	− 0.273***	0.21**	–						
Compassion	0.114	0.07	0.126	0.1	0.102	0.026	0.04	0.147*	0.057	–					
Amusement	0.113	0.094	− 0.037	0.012	0.144*	0.105	− 0.044	0.082	0.065	0.107	–				
Love	0.025	− 0.085	**0**.144*	0.111	0.01	− 0.121	− 0.114	0.046	0.455***	0.245***	− 0.006	–			
Pride	0.056	− 0.178*	0.098	0.012	0.135	− 0.113	− 0.165*	**0**.199**	0.672***	0.061	− 0.074	**0**.415***	–		
AWE	0.186**	− 0.041	0.283***	0.122	0.126*	0.040	0.035	**0**.253***	0.389***	**0**.223**	**0**.155*	**0**.158**	0.155*	–	
Reappraisal	**0**.193**	0.133	0.187**	0.216**	0.058	0.037	0.035	0.188**	0.321***	0.206**	0.033	0.168*	0.375***	0.268***	–
Suppression	0.127	0.204*	− 0.02	0.125	0.005	0.152*	0.167*	0.021	− **0**.349***	0.045	0.048	− 0.395***	− 0.206**	− 0.07	**0**.179*

*p < 0.05, **p < 0.01, ***p < 0.001.

### Awe triggers

**Figure 2:**
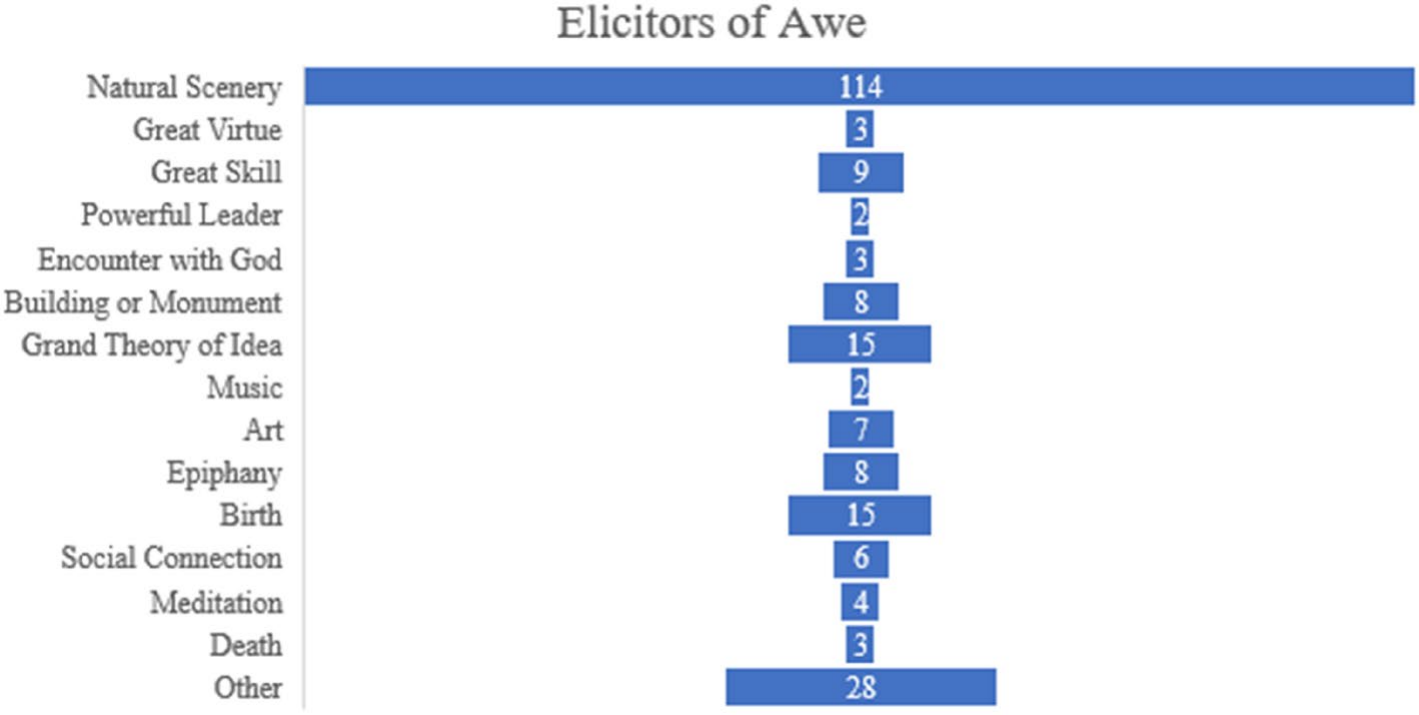
Funnel chart showing frequency of triggers of Awe in this sample. N = 227.

A coder expert in awe research was supplied with the written excerpts and then asked to extract the main elicitor of awe described in the excerpt, starting from categories used in Yaden et al.^23^ and listed in the Keltner and Haidt’ seminal model of awe^1^. Each excerpt was then coded according to the elicitor. “Social Connection” was added as an additional category, given also recent findings on the social elicitors of awe^3^ (Fig. 2).

**Figure 3:**
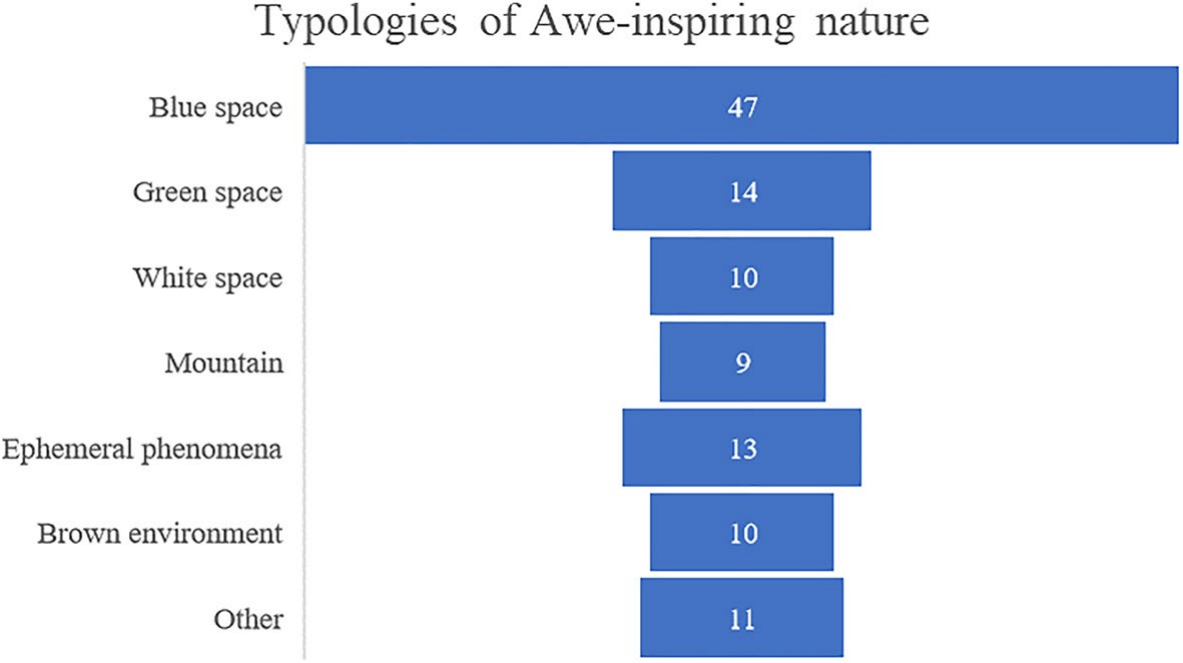
Funnel chart showing frequency of categories of awe-inspiring nature in this sample. N = 114. We identified these typologies of Nature: Blue spaces^77^; Green spaces^78^; White spaces^79^; Ephemeral phenomena (sunset, sunrise, and storm)^80^; Brown environments^81^; Mountains (when participants reported the specific elicitor but not its features, e.g., whether it was green, white, or brown). We also added the category ‘Other’ for those natural environments whose features were not clearly reported by participants.

Moreover, we also identified these typologies of Nature: Blue spaces^77^; Green spaces^78^; White spaces^79^; Ephemeral phenomena (sunset, sunrise, and storm)^80^; Brown environments^81^; Mountains (when participants reported the specific elicitor but not its features, e.g., whether it was green, white, or brown). We also added the category ‘Other’ for those natural environments whose features were not clearly reported by participants (Fig. 3).

## Study 2

### Participants

The study included 106 participants (74 females—mean age = 27.2; S.D. = 9.2; males − mean age = 29.6; S.D. = 11,3). They were adults over 18, mostly educated (4.72%, n = 5 of them had a middle school diploma, 24.53%, n = 26 presented a high school diploma, 51.89%, n = 55 had a bachelor’s degree and 18.87%, n = 20 had a master’s degree). Moreover, 39.7% (n = 42) of participants reported having previous experience with VR.

Participants were involved in the same awe-inspiring training in a social virtual reality platform called AltspaceVR, in which all the scenarios were preliminarily tested for their effectiveness to elicit awe. Participant underwent the online training and finally, they completed the AWE-S on Qualtrics. The experimental protocol was approved by the Ethical Committee of the Università Cattolica del Sacro Cuore prior to data collection. Each participant provided an electronic informed consent for study participation. Participants’ consent and all methods were carried out in accordance with the Helsinki Declaration.

### Measures

#### Italian version of the awe experience scale (AWE-S)

The Awe Experience Scale^23^ validated in the previous study (study 1) in Italian was used in this study.

#### Italian validated version of the dispositional positive emotions scale (DPES)—awe subscale.

In the two studies, again, the Italian validation^4^ of the Dispositional Positive Emotions Scale^71^- Awe Subscale was used.

#### ITC-SOPI sense of presence inventory

It is a well-validated questionnaire composed of 42-items on a 5-point Likert scale (1 = strongly disagree; 5 = Strongly agree). We used the Italian adapted version of this questionnaire^82^. This questionnaire consists in four subscales, each referring to a specific dimension of presence, with a good original internal consistency (Cronbach Alpha ranging between 0.76 and 0.94): Sense of Physical Space (0.94); Engagement (0.89); Ecological Validity (0.76); Negative Effects (0.77).

### Procedure

Participants underwent an interactive screen-based validated awe-inspiring training on the social virtual platform *AltspaceVR,* which had been previously tested^56^ and consisted of four phases*.* The first two phases served as tutorial and introductory functions. As a third step, participants accessed either the validated virtual world “Deep space”, or the VR scenario “Tall Forest”, both featuring an awe-inspiring narrative lasting about 5 min. In the last phase, participants were administered the AWE-S, the ITC-SOPI Sense of Presence Inventory, and DPES. The entire training lasted one hour with 3 different moments of pause for participants to minimize possible fatigue and cybersickness.

To detail the training, a brief description of each phase is provided. The training was divided into four phases. On the day before the training, participants were invited to download AltspaceVR on their own laptops. Participants used AltspaceVR’s non-immersive but interactive format. No headset was used, and they navigated and interacted with objects using their keyboard. The participants were trained in AltspaceVR use on the day of the training. The facilitator demonstrated and explained basic controls (e.g., walking, turning on a microphone, listening to other participants, etc.), and participants were given the opportunity to try them. Then, participants were instructed to open the link that contains direct access to the AltspaceVR awe-inspiring world. After a brief introduction on emotion science (elicitors, appraisal themes, action tendencies, evolutionary function) using the virtual presentation provided by the VR platform, participants accessed the "Deep Space" world, which featured a validated awe-inspiring narrative lasting about 5 min. The facilitator determined the beginning and end of the exposure. The participants were then required to proceed to the second scenario, Tall Forest, where another validated five-minute awe-inspiring narrative began. Both scenarios were accessed by all participants in a counterbalanced order, according to the instructions provided by the facilitator. At the end of the experience, participants were invited to participate in two different activities. They were invited to share the experience and then to make associations between the current moment and their daily lives. Finally, AWE-S was administered, along with ITC-SOPI and DPES.

If we assumed that the same structure of AWE-S as in Italian Study 1, along with the original autobiographical instructions, could also be found in Study 2 with a standardized induction of awe (i.e., just exposing participants to a VR scenario, without prompting them with an explicit description of awe), we would have achieved a first promising step towards identifying this emotion' semantic domain.

## Results

### Preliminary data analyses

We carried out a CFA to test the fit of the Italian AWE-S derived in the previous study. To analyze this 29-item 6-factor model for CFA we used MPLUS software 8^72^, computing also Comparative Fit Index (CFI), Tucker–Lewis index (TLI), and Root Mean-Square Error of Approximation (RMSEA), and standardized Root Mean Square Residual (SRMR), AIC (Akaike Information Criterion) and BIC (Bayesian Information Criterion) to evaluate the differences between the various models tested^83^. Preliminarily, we checked for normality. Data was not distributed normally. No multivariate outliers were identified.

The determination of model fit was based on a comparison of the fit indices obtained from the 4 CFAs with the suggested cutoff values frequently cited in the literature for the CFI, TLI, RMSEA, and SRMR indices (c.f.^84^). According to Schreiber et al.^83^, A RMSEA below of 0.08 is an acceptable fit; a Tucker–Lewis Index (TLI) greater than 0.90 indicates is an acceptable fit; Comparative Fit Index (CFI) greater than 0.90 indicates is an acceptable fit; Standardized Root Mean Square Residual (SRMR) less than 0.08 indicates is an acceptable fit.

Default settings in the CFA model are as follows: (i) the factorial saturation of the first variable of each factor is set to 1, (ii) covariances between residuals are set to 0, (iii) factor variances are estimated, (iv) covariances among the exogenous variables are estimated and the estimation method is maximum likelihood mean adjusted (MLM). MLM was used since it is robust regarding not normal data distribution.

Further model revisions based on modification indices were computed. CFA with 106 participants was conducted for the 6 factors, with no missing values. The Italian version has the same structure as the English one, and the first model (FULL model) is a reproduction of it. The full CFA model yielded ambiguous results: χ2(362) = 687.532, *p* < 0.001; RMSEA = 0.092, 90% CI = 0.082 0.103, *p* < 0.001; CFI = 0.899; TLI = 0.887; SRMR = 0.081). The model has fitness issues; however, the standardized betas are still high. The modification indexes suggested the need to force some correlations between some items, which were implemented one at a time in three successive models, with the last one being the best for fitness and beta standardized. Here we present the fitness indexes in the four models (full model, “M1” model with correlation between items 27–28, “M2” model with correlation between item 27–28 and 24–22, “M3” model with correlation between items 27–28, 24–22 and 14–12, “M4” model with correlation between items 27–28, 24–22, 14–12 and 25–21). See Table 3. For CFA summary of results. When we assumed that the factors correlated, the confirmatory structure was adequate.

**Table 3:** CFA summary of results.

	AIC	BIC	SRMR	RMSEA	CFI	TLI
Full model	8862.7	9134.4	0.081	0.092	0.899	0.887
M1	8787.1	9061.4	0.057	0.083	0.919	0.909
M2	8683.7	8960.7	0.062	0.067	0.947	0.94
M3	8666.8	8946.4	0.061	0.064	0.952	0.946
**M4**	**8649.8**	**9134.4**	**0.057**	**0.061**	**0.957**	**0.951**

N = 106; **in bold** the final best model.

We computed correlations between “Awe” factor of DPES and all AWE-S subscales. The Awe DPES factor showed medium significant correlations with some AWE-S dimensions [Time (r = 0.216; *p* < 0.01); Self-Loss (r =− 0.194; *p* < 0.01); Connection (r = 0.262; *p* < 0.01)], but not with others [Vastness (r = 0.150; *p* > 0.05); Physical sensations (r = 0.092; *p* > 0.05); Need for accommodation (r = 0.137; *p* > 0.05)].

We also computed Pearson’s correlations between ITC-SOPI sense of presence inventory dimensions (Sense of Physical Space, Engagement, Ecological Validity, Negative Effects) and AWE-S subscales. Ecological Validity showed negative significant correlations with three AWE-S subscales, namely, Time (r = − 0.223; *p* < 0.05), Connectedness, (r = − 0.254; *p* < 0.01), Physical Sensations (r = − 191; p < 0.05).

Internal consistency for each factor computed as Cronbach Alpha coefficient was high: Time (α = 0.956); Self-loss (α = 0.970); Connectedness (α = 0.960); Vastness (α = 0.955); Physical Sensations (α = 0.891); Need for Accommodation (α = 0.890). Finally, we computed internal correlations among AWE-S factors (see Table 4).

**Table 4:** Pearson’s correlations among AWE-S factors.

	Time	Self-loss	Connection	Vastness	Physical sensations	Need for accommodation
Time	–					
Self-loss	0.664***	–				
Connection	0.503***	0.443***	–			
Vastness	0.503***	0.568***	0.69***	–		
Physical sensations	0.345***	0.371***	0.602***	0.638***	–	
Need for accommodation	0.422***	0.416***	0.199*	0.263**	0.235*	–

*p < 0.05, **p < 0.01, ***p < 0.001.

## Discussion

The AWE-S was translated from English to Italian. The latent structure of the AWE-S was examined in two studies: first, from autobiographically eliciting awe, and then from an awe-inspiring virtual reality experience. In addition to providing a translation, a major purpose of these studies was to determine differences in the experience of awe when compared to two different approaches to eliciting emotions. After a preliminary validation study was conducted in Italian, the resulting translation was tested in the context of a virtual reality study. The overall structure of the original English AWE-S was replicated in Italian, both in the context of an autobiographical recall experience of awe and as part of a standardized elicitation of awe using virtual reality.

Specifically, a robust 6-factor structure for the Awe Experience Scale (AWE-S), as a state measure of awe that was validated and used in English-speaking countries, held in an Italian context both during an autobiographical recall study (Study 1), as well as in a VR study (Study 2). In Study 1, an EFA on 30 original items administered to online participants (n = 350), revealed a 6-factor structure. Only one item (29 item) was dropped due to extremely low communality, and it did not load on any factor. The 6-factor structure held, with good reliability for each factor.

In Study 2, the novel 29- item AWE-S Italian scale was administered to participants (n = 106) by inviting participants to navigate two virtual reality awe-inspiring scenarios, already validated in a previous study^56^. CFA confirmed a robust fit of the 6-factor model. In general, a 6-factor solution with high internal reliability was found both in Study 1 and in Study 2. In Study 1, to compute convergent, divergent, and construct validity, the Italian AWE-S was compared with two trait measures, i.e., the Italian DPES, the Italian version of the ERQ, and one state-measure of affect, i.e., the PANAS.

At the trait level, the DPES is a dispositional measure of one’s proneness to live a number of different positive emotions and it was the only available Italian validated scale directly measuring awe, although it taps into a trait level of awe. In study 1, Time, Connection and Physical Sensations dimensions of AWE-S significantly and positively correlated with the Dispositional Awe subscale of DPES, thus, partially supporting a convergent validity with the trait Italian awe measure during the autobiographical recall. However, in study 2 with VR, a significant negative correlation between DPES Awe subscale and Self-loss and a significant positive correlation between DPES Awe subscale and Time together with Connection emerged, while the positive significant correlation with Physical Sensations (AWE-S dimension) was lost. On the other hand, Self-loss featured a significantly negative correlation with dispositional Awe only during the VR training, and not while recalling the experience. Moreover, the physical sensations dimension was strongly correlated with DPES Awe subscale during autobiographical recall but not in VR exposure, thus suggesting that the body can play a different role during a standardized exposure in simulated environments, maybe, also able to overcome individual differences. At the same time, Vastness was also not found to be associated with the trait measure of awe. Dimensions of Time and Connectedness, usually belonging to self-transcendence domain, preserved positive correlations with the dispositional measure of awe both in autobiographical recall and in VR. Moreover, correlations between another trait measure, the ERQ, and AWE-S dimensions during autobiographical recall (Study 1) were significant and positive for Time, Connection and Vastness, which can suggest the need for an increased cognitive processing of the original elicitor. While suppression—i.e., the other emotion regulation strategy—showed significant positive correlations with Self-loss and Need for accommodation dimensions. This might be due to the intensity of the emotional experience, which turned into a cognitively high demanding phenomenon both at the level of self-perception, as well as in terms of the overall experience. Indeed, it was showed that situational induction of awe were related with greater uncertainty^85^, and, actually, awe can make what is unknown or uncertain salient and potentially approachable^86^. However, it should be still deepened how this mechanism works in terms of emotion regulation in autobiographically recalled situational awe. For instance, a previous study showed that benefits from awe-inspiring videos and dispositional awe in terms of wellbeing during awaiting periods, were not significantly higher than those obtained from being exposed to general positive videos^87^.

While these convergent relationships were quite low, this might be expected when comparing a trait measure with a state measure. In some studies, for example, dispositional awe has been found to play a significant role in shaping specific prototypical experiences of awe^88^, but in others this relationship was not observed^89^. Conversely, since autobiographical recall depends on individuals’ specific memory recall abilities^90^, it might be related to individual ability to experiencing awe. Individuals do not construct memories of past and future events in isolation from other aspects of their lives, as autobiographical memory refers to our personal self-narrative and the broader significance of events in relation to our overall lives^90^. Thus, another hidden factor that might be assumed to play a role in awe elicited by autobiographical recall (vs. VR) can be the *personal relevance* associated to the elicitor itself.

At the state level, regarding PANAS dimensions of Positive and Negative Affect some significant correlations with AWE-S dimensions were found. Again, Self-loss positively correlated with Negative Affect, thus confirming the negative nuance associated with this AWE-S dimension, as it has already emerged for the Suppression factor for ERQ. Specifically, For the ERQ factors, this study 1 found that Reappraisal correlated positively with almost all dimensions of AWE-S, as did Suppression, with “Need for Accommodation” and “Small Self” dimensions, suggesting that this experience is not simply a positive or negative emotion, rather both sides of valence emerged to be embedded in it.

Consistently, regarding DPES, significant negative correlations between Self loss and Happiness and Pride factors emerged. Conversely, Negative Affect significantly and negatively correlated with Connection and Vastness, as the possible positive counterparts of the Self-loss dimension. These results could suggest that some dimensions of awe experience pertain more to the negative side of awe, while others to the positive ones, thus bringing forth the mixed nature of this emotion^7,43^. Future studies may additionally consider state qualities that fall between the purely cognitive and affective elements, such as mental flexibility^91,92^. Indeed, a nonlinear combination of cognitive and affective factors may have an impact on an experience as complex as awe. Our ability to operate adaptively and variably in response to environmental pressures, or mental flexibility, may have an impact on how we experience awe. For instance, extremely rigid people might not be able to comprehend the various dimensions of the concept of awe.

In terms of the specific factors of awe in the AWE-S, the original English structure of the scale held both for Autobiographical recall as well as for VR induction, but with some peculiarities. The original English item 29, tapping into the need for accommodation dimension was dropped to low communality and since it did not load in any factor. This is not surprising since this dimension has always been difficult to operationalize^74^. Going forward, novel studies exploring the role of expectation violation and exceed expectations^93^ could be used to inform the definition of this construct.

Finally, concerning the link between sense of presence and each dimension of AWE-S, it is noteworthy that only three significant negative correlations were found. Specifically, the more the VR scenario resembled an equivalent real one, the less participants experience a sense of time distortion, connectedness and physical sensations usually associated with a typical awe experience. It might be that participants’ awe experience in a non-immersive environment—compared to an immersive an interactive one^90^—was mainly driven by the paradoxicality of the VR scenarios, which featured the view of the Earth from outside its atmosphere^94^ and an enchanted forest. Magic is usually associated with awe^95^, although no systematic analysis of their link has been conducted so far.

Finally, regarding awe elicitors (Study 1), different types of natural stimuli still emerged amongst the most frequent triggers of awe as in^23^, while also conceptual inductors were curiously frequently reported. Specifically, in this sample, conceptual elicitors, also including moments of “introspection” resulted in higher occurrence. Even more unusually, social elicitors – especially, in close relationships—clearly emerged as amongst awe’s triggers in this sample of the Italian population. These discoveries can advance cross-cultural and ecological inquiry on awe’s elicitors.

## Limitations and future directions

This study has several limitations. First, the Italian translation of the specific term “awe” by consultation among experts gave rise to a periphrasis in which a noun and an adjective were combined (it. *profonda meraviglia*)^70^ and a description of the characteristics of this experience was provided to participants in the autobiographical recall study, to replicate the exact instructions of the original English scale^23^. As mentioned in Study 2, upon assumption that the Italian Study 1 structure of AWE-S, with the original autobiographical instructions would also be found in study 2 with a standardized induction of awe (the same stimuli for all participants, not accompanied with a description of awe experience), we would have made a first promising step toward elucidating the semantic domain of this emotion. In fact, the AWE-S does not explicitly mention the word "awe", therefore, participants in study 2 could not anchor any hints when answering the questionnaire. Both studies confirmed the model’s suitability.

Another limitation derived from the current lack of a validated state measure of awe in the Italian research literature that would allow us to better establish convergent and divergent validity. A Dispositional measure of awe has been validated and it was included here, but some studies have shown that awe can act differently at the trait and at the state level. Moreover, there was a gender imbalance within our samples, with more female respondents than male respondents. The potential impact of this variable on the experience of awe should be examined in future studies.

Furthermore, to deepen the analysis of the potential of autobiographical recall and VR on awe elicitation, it would be useful to also include a measurement of sense of presence. We assessed the sense of presence in the simulated environments of the VR-based awe-inspiring training, but we did not consider this variable in relation to participants’ ability to simulate past memories in a vivid way.

In conclusion, awe has been conceptualized in a variety of ways (despite being interrelated). As a result, even though Keltner and Haidt's conceptualization of awe has received significant attention, especially in experimental research, future studies should consider different operationalization or definitions of this emotion (e.g.,^23,24,28^), including those from other disciplines, such as philosophy (e.g.,^24,96,97^).

## Conclusions

This series of studies showed a high degree of overlap between the overall experience of awe through the classical autobiographical recall technique as well as a more standardized induction of this emotion using VR. The global structure of the experience held in both situations but potentially cultural variations at the state and at the trait level were observed, thus suggesting to deepening the analysis of how this experience unfolds in non-English speaking countries and how it is associated to the tendency to live such experience. Specifically, this scale can be further used to unveil dimensions of awe possibly responsible for its negative and positive nuances. Despite the fact that the 6-factor model held for both emotion-elicitation techniques, more work is needed to investigate the lexical aspects of awe in non-English speaking countries.

## Data Availability

The datasets used and/or analyzed during the current study can be made available from the corresponding author on reasonable request.
