# Author Correction: Unveiling the underlying structure of awe in virtual reality and in autobiographical recall: an exploratory study

**DOI:** 10.1038/s41598-024-69305-7

**Published:** 2024-08-06

**Authors:** Alice Chirico, Francesca Borghesi, David B. Yaden, Marta Pizzolante, Eleonora Diletta Sarcinella, Pietro Cipresso, Andrea Gaggioli

**Affiliations:** 1 Department of Psychology, Research Center in Communication Psychology, Università Cattolica del Sacro Cuore, Milan, Italy; 2 Department of Psychology, University of Turin, Via Verdi 10, 10124 Turin, Italy; 3 Department of Psychiatry and Behavioral Sciences, Johns Hopkins University School of Medicine, Baltimore, MD USA; 4 IRCCS Istituto Auxologico Italiano, Milan, Italy

Correction to: *Scientific Reports* 10.1038/s41598-024-62654-3, published online 30 May 2024

The Funding section in the original version of this Article was incomplete.

“This work was supported by Italian Ministry of Health—Ricerca Corrente; the Ministry of University and Research (MUR), formerly Ministry of Education, University and Research (MIUR) within a project funded by “Education and research for recovery—REACT-EU”, included in the NOP Research and Innovation 2014–2020, by PON R&I 2014-2020 (FSE REACT-EU); and by the grant PRIN 2022NWRENN - E-MOTIONS funded by the Italian Ministry of Research.”

now reads:

“This work was supported by Italian Ministry of Health—Ricerca Corrente; the Ministry of University and Research (MUR), formerly Ministry of Education, University and Research (MIUR) within a project funded by “Education and research for recovery—REACT-EU”, included in the NOP Research and Innovation 2014–2020, by PON R&I 2014-2020 (FSE REACT-EU); and by the grant PRIN 2022NWRENN - E-MOTIONS funded by the Italian Ministry of Research. This study was partly supported by the BIAL Foundation (SUBRAIN project 'The origin of the sublime power in the brain: an integrated EEG-TMS study', Grant n° 288/20 to AC).”

The original Article has been corrected.

